# Can a Proper T-Cell Development Occur in an Altered Thymic Epithelium? Lessons From EphB-Deficient Thymi

**DOI:** 10.3389/fendo.2018.00135

**Published:** 2018-04-03

**Authors:** Juan José Muñoz, Javier García-Ceca, Sara Montero-Herradón, Beatriz Sánchez del Collado, David Alfaro, Agustín Zapata

**Affiliations:** ^1^Center for Cytometry and Fluorescence Microscopy, Complutense University of Madrid, Madrid, Spain; ^2^Department of Cell Biology, Faculty of Biology, Complutense University of Madrid, Madrid, Spain

**Keywords:** thymus, thymocytes, thymic epithelial cells, Eph, ephrins

## Abstract

For a long time, the effects of distinct Eph tyrosine kinase receptors and their ligands, ephrins on the structure, immunophenotype, and development of thymus and their main cell components, thymocytes (T) and thymic epithelial cells (TECs), have been studied. In recent years, the thymic phenotype of mutant mice deficient in several Ephs and ephrins B has been determined. Remarkably, thymic stroma in these animals exhibits important defects that appear early in ontogeny but little alterations in the proportions of distinct lymphoid cell populations. In the present manuscript, we summarize and extend these results discussing possible mechanisms governing phenotypical and functional thymocyte maturation in an absence of the critical T–TEC interactions, concluding that some signaling mediated by key molecules, such as MHCII, CD80, β5t, Aire, etc. could be sufficient to enable a proper maturation of thymocytes, independently of morphological alterations affecting thymic epithelium.

## Introduction

The organogenesis of complex tissues requires the coordinated differentiation of cells at the correct time and place. A central role for Eph kinase receptors and their ligands, ephrins in these processes, has been claimed (1, 2) and, particularly, in the thymus (3). Both Eph and ephrins provide positional information for cells, regulating cell-to-cell contacts, cell migration, cell survival and differentiation. Eph receptors include EphA (10 members) that preferentially, but not exclusively, bind ephrins-A (6 members) and EphB (6 members) that interact with ephrins-B (3 members). In this system, Eph/ephrin binding results in a bidirectional signaling, forward in the case of Eph and reverse in that of the ephrins (4). Both molecules that partially govern the establishment of the neural network in the central nervous system (5) also modulate the thymocyte migration throughout the thymic compartments (6) and temporal and topological thymocyte (T)–thymic epithelial cell (TECs) interactions (3, 7). The relevance of such cell-to-cell interactions has been classically recognized, but some recent data question its importance (8). In the present analysis, we limit the relevance of T–TEC crosstalk to explain the absence of a clear thymocyte phenotype in thymi exhibiting a severely altered thymic epithelial network.

## The Thymic Phenotype of EphB-Deficient Mice

Phenotypes of thymocytes and TECs are remarkably different in EphB2 and EphB3 knockout thymi. Three major features characterize these thymi: hypocellularity, profound alterations in the morphology and histology of TECs, and few changes occurring in the proportions of distinct thymocyte subpopulations. In addition, these phenotypes appear early in the thymus ontogeny and gradually increase when T–TEC interactions become more intense in WT thymi.

### Absence of EphB2 and/or EphB3 Courses With Low Number of Both Thymocytes and TECs

The lack of Eph or ephrins courses with thymic hypocellularity that affect both thymocytes (9, 10) and TECs (11, 12), and the blockade of Eph/ephrin signaling reduces thymic cell numbers (13, 14).

Low numbers of recent emigrants seeding the mutant thymi and their slow maturation (10, 15) are a major cause of the thymic hypocellularity, in addition to their increased apoptosis and reduced proportions of cycling thymocytes (9). Reduced proportions of cycling thymocytes could be associated with decreased Delta-like 4 (Dll4) and IL7 receptor α chain transcript (12) as is also observed in thymocytes exhibiting specific deletion of ephrin-B1 and ephrin-B2 (16), both molecules involved in the maturation of developing double-negative (DN) thymocytes (17, 18).

Reduced lymphoid immigrants affect, by turn, the proportions of immature MTS20^+^ TECs (10) contributing to their delayed maturation and decreased TEC numbers. Also, altered proportions of cycling TECs and apoptotic TECs account for TEC hypocellularity of mutant thymi (12). Fetal and postnatal thymi of EphB2- and/or EphB3-deficient mice show higher proportions of apoptotic TECs than WT ones, which correlates with a reduced thymic K8^+^ epithelial network (19). At E12.5, cell proliferation is delayed in Eph-deficient TECs as a consequence of the delayed seeding of lymphoid progenitor cells into mutant thymic primordium (10, 12). Later, decreased proportions of cycling cells, which could be partially related to decreased transcripts of FGF7 and its receptor FGFR2IIIb (12) involved in thymic epithelium proliferation (20, 21), have been related with the delayed maturation of mutant thymic epithelium.

### Delayed Maturation of TEC Subsets Also Occurs in EphB-Deficient Thymi

Important morphological and immunohistochemical changes occur in the epithelial cell subpopulations of EphB-deficient thymi, including the presence of K5^+^K8^+^MTS10^+^ immature medullary TECs (mTECs), high numbers of K5^−^K8^−^MTS20^+^ cells and K5^+^K8^+^ cortical TECs (cTECs) and increased number and size of K5^−^K8^−^ epithelial-free areas (11, 22). By flow cytometry, we confirmed delayed maturation of immature MTS20^+^ TECs, cTECs defined by the expression of Ly51, CD205, MHCII, CD40 and β5t, and mTECs identified by UEA-1, Cld3/4, SSEA-1, MHCII, CD40, CD80, and Aire medullary markers (12, Montero-Herradón et al 2017, submitted manuscript)^1^. This defective epithelial maturation culminates in the aberrant phenotypes of mutant adult thymi, in which the 3D epithelial network is disrupted by the inability of thymocytes and TECs to adequately intermingle.

On the other hand, although it has been reported that the absence of one or several Eph has no phenotype because of the known promiscuity of this molecular system, in which practically any Eph and ephrin can interact thus favoring a certain overlapping (23, 24), we recently demonstrated a specificity in the effects of EphB2 and EphB3 on TECs. Remarkably, although both EphB2 and EphB3 are necessary for a proper development of both cortical and medullary epithelia, the lack of EphB2 results in a more severe medulla phenotype than that of EphB3^−/−^ mTEC (Montero-Herradón et al 2017, submitted manuscript)^1^, whereas the thymic cortex of EphB3^−/−^ mice is particularly affected (12).

### Morphological Changes in the Mutant TECs

**Figure 1:**
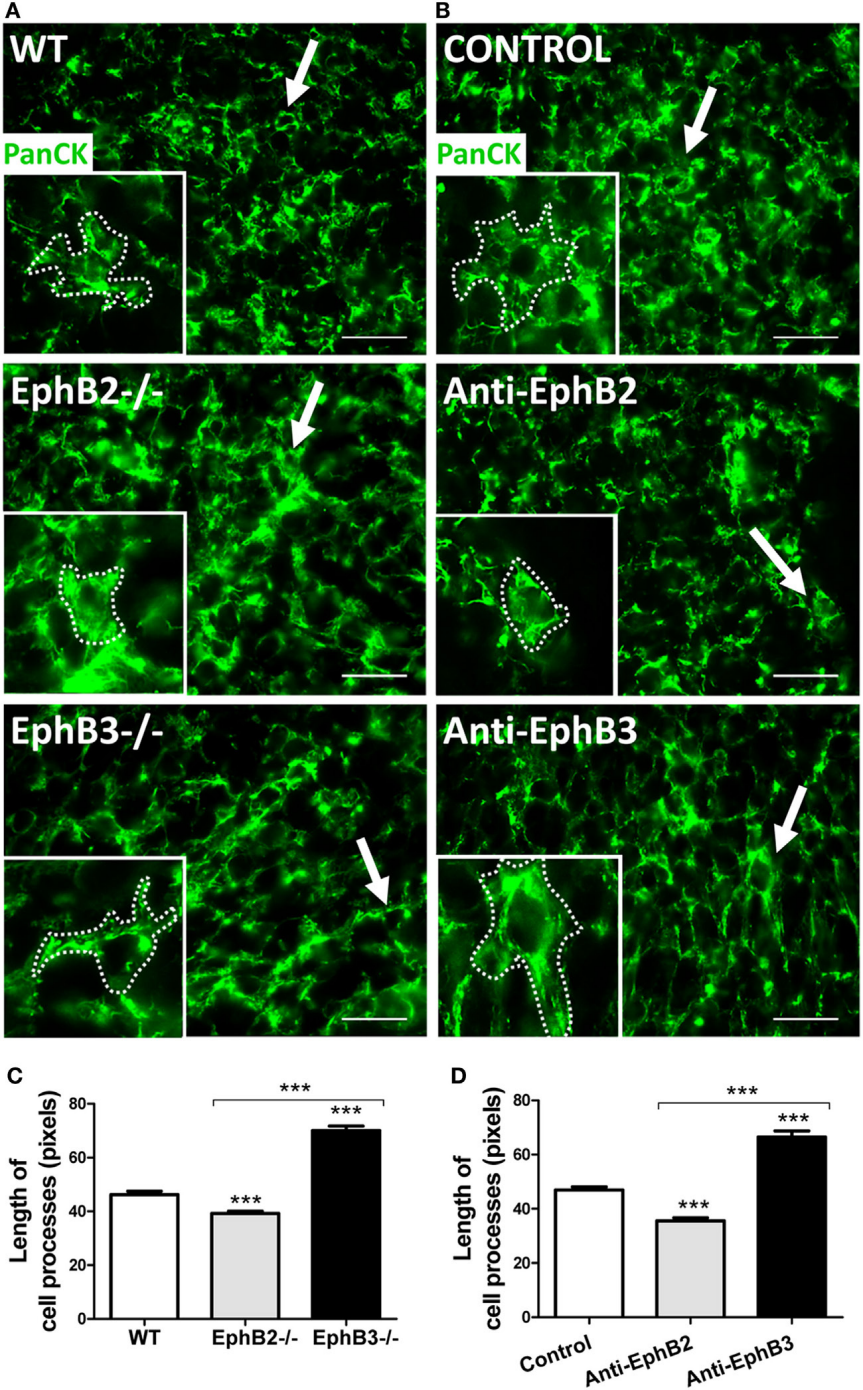
Thymic epithelial cell (TEC) morphology in reaggregate thymus organ cultures (RTOCs) established with either WT cells or EphB-deficient cells, or RTOCs treated with either blocking anti-EphB2 or anti-EphB3 antibodies. **(A,B)** Standard immunofluorescence study of the TEC network stained with an anti-PanCytokeratin antibody (PanCK, Green) and details of the TEC morphology in the different established RTOCs. Notice the shortened epithelial cell processes (arrows and insert dotted line) in RTOCs established with EphB2^−/−^ cells **(A)** or treated with a blocking anti-EphB2 antibody **(B)** and the elongated cell processes (arrows and insert dotted line) in RTOCs established with EphB3^−/−^ cells **(A)** or treated with anti-EphB3 antibody, as compared with their respective WT controls **(A)** or isotype control antibodies [**(B)**, control]. The inserts illustrate the morphology of these cells. Scale: 50 µm. **(C,D)** Morphometric analysis of the length of cell processes in RTOCs established with either EphB2- or EphB3-deficient cells or RTOCs treated with blocking anti-EphB2 or anti-EphB3 antibodies. Note the reduced length of cell processes in RTOCs established with EphB2^−/−^ cells **(C)** or treated with anti-EphB2 antibody **(D)**, while those established with EphB3^−/−^ cells **(C)** or treated with anti-EphB3 antibody **(D)** show longer cell processes as compared with their control RTOCs. The length of cell processes was measured in pixels in those cells whose cell body appeared sectioned. Five RTOCs of each experimental group were studied measuring about 25 cells and a total of 100–150 cell processes by reaggregate. The significance of the Student’s *t*-test probability is indicated as ***p ≤ 0.005.

The absence of EphB specifically affects the TEC morphology. In the medulla, mTECs undergo a shortening of cell processes appearing as globular cells in both EphB2^−/−^ and EphB3^−/−^ thymi, but in the cortex EphB2^−/−^ cTECs show reduced cell processes resulting in a rounded cell shape, whereas EphB3^−/−^ cTECs exhibit long, perpendicular cell processes. Independently of the changes undergone, mutant cells appear considerably separated from both thymocytes and other TECs (11). In order to confirm whether the lack of either EphB2 or EphB3 affected TEC shape, reaggregate thymus organ cultures (RTOCs) formed from WT fetal thymus lobes treated (or not) with either blocking anti-EphB2 or anti-EphB3 antibodies were examined. In treated RTOCs, TEC morphology was similar to that of the respective mutant thymi: rounded in those treated with anti-EphB2 and exhibiting long, perpendicular cell processes in those receiving anti-EphB3 (Figures 1A,B). In these conditions, epithelial cell processes were significantly shorter in both EphB2^−/−^ or anti-EphB2-treated RTOCs and longer in EphB3^−/−^ and anti-EphB3-treated RTOCs, than in WT RTOCs (Figures 1C,D).

It is largely known that the Eph/ephrin signaling modulates cytoskeleton and cell adhesion (25). More specifically, in RTOCs, the blockade of Eph signaling by soluble ephrin-B1Fc provokes TEC rounding with the disappearance of cell processes and disorganization of the cytoskeleton (13). In agreement, EphB2 and EphB3 regulate the morphology of neuronal dendrite spines (26), the lack of ephrin-B2 elongates muscle cells and induces lamellipodium formation (27), and bone marrow-derived mesenchymal stromal cells treated with EphB2-Fc or EphB4-Fc fusion proteins undergo roundness and reduced size (28). It is evident, therefore, that morphological changes in TECs of EphB-deficient thymi hinder the establishment of proper cell-to-cell contacts between thymocytes and TECs, critical for the adequate maturation of both thymic cell components, as previously indicated for the increased apoptotic TECs found in mutant thymi.

### Histological Organization of Thymic Cortex and Medulla

In the thymic medulla of both fetal EphB2- and EphB3-deficient thymi, there are profound modifications that after birth, they remain in EphB2^−/−^ medulla and improve partially in EphB3^−/−^ thymi. Mature thymic medulla is organized from individual islets that expand and fuse after birth (29). By contrast, in mutant thymi, particularly in EphB2^−/−^ ones, a unique adult thymic medulla is impaired and only small isolated foci remain. Adult EphB3-deficient thymi have a more organized central medulla but small, scattered medullary foci also appear (11). Furthermore, the quantification of these medullary foci in RTOCs established again with either WT, EphB2^−/−^, EphB3^−-/−^, blocking anti-EphB2- or anti-EphB3-treated fetal thymus lobes confirmed the existence of more and significantly smaller foci in mutant and treated lobes, with differences between those deficient in EphB2 and EphB3 (Montero-Herradón et al 2017, submitted manuscript)^1^.

## The Condition of T Cells in EphB-Deficient Mice

Although lower numbers of lymphoid progenitor cells seed both mutant adult and embryonic thymi than in WT mice (10, 15), their subsequent progression does not result in notable changes in the percentages of distinct thymocyte subsets. Some delayed maturation of the DN cell subpopulations occurs with increased proportions of total DN cells and unchanged values of both double-positive (DP) cells and single-positive thymocytes. Within the DN cell populations, DN1 cells increased, whereas DN3 cell compartment underwent a significant reduction (9, 10). Decreased proportions of DN3 thymocytes could be associated with changes in TCR selection or molecules involved in their maturation, such as Dll4 and IL7 whose transcripts diminish in EphB-deficient thymi (12). In addition, Luo and colleagues (16) reported a reduced expression of the IL7 receptor α chain in ephrin-B1/ephrin-B2-deleted thymocytes.

On the other hand, analysis of the TCR repertoire of mutant CD4^+^ cells by using a battery of antibodies specific to different TCR rearrangements only found increased proportions of Vβ3^+^CD4^+^ cells in both thymus and lymph nodes (30), and the peripheral lymphoid organs (peripheral blood, spleen, lymph nodes) did not exhibit an altered architecture, a disturbed topological distribution of lymphoid and macrophage areas (30) nor significant changes in the proportions of CD4/CD8 T lymphocyte subpopulations (9). Presumably, the mesenchyme-derived stroma of both spleen and lymph nodes is less affected than TECs by the lack of EphB. On the other hand, no changes occur in the proportions of TH1 (TCRαβ^+^CD4^+^IFNγ^+^), TH2 (TCRαβ^+^CD4^+^IL4^+^), and TH17 (TCRαβ^+^CD4^+^IL17^+^) cells between mutant and WT mice in either spleen or inguinal lymph nodes. Nor do the proportions of splenic TCRαβ^+^CD4^+^CD25^+^Foxp3^+^ Treg change when values of EphB2^−/−^ (0.83 ± 0.25), EphB3^−/−^ (0.72 ± 0.16), and WT mice (0.96 ± 0.27) are compared. By contrast, Treg of inguinal lymph nodes show significantly higher values in EphB2^−/−^ (4.29 ± 0.40) and EphB3^−/−^ mice (4.23 ± 0.44) than in WT ones (3.89 ± 0.45).

Recently, we evaluated other lymphoid cell populations particularly those involved in thymocyte selection within the thymus. No differences occurred in either positive selected TCRαβ^hi^CD4^+^CD8^+^CD69^+^ and TCRαβ^hi^CD4^+^CD8^−^CD69^+^ thymocytes in both EphB2- (3.00 ± 0.61; 8.00 ± 1.91) and EphB3 (2.17 ± 0.40; 6.60 ± 1.73)-deficient thymi, as compared to WT values (2.47 ± 0.66; 7.09 ± 3.00). Nor did the percentage of both total TCRαβ^hi^Foxp3^+^ and TCRαβ^hi^CD4^+^Foxp3^+^ regulatory T cells (Treg) change when EphB2^−/−^ mice (0.94 ± 0.06; 0.69 ± 0.10) and WT ones (0.90 ± 0.18; 0.66 ± 0.12) were compared, although values in EphB3^−/−^ thymi were slightly lower, but not significantly, than in the other two mice analyzed (0.68 ± 0.07; 0.54 ± 0.04).

Negative selection was evaluated in WT and EphB-deficient mice comparing the proportions of total caspase3^+^CD5^+^CD69^+^CD4^+^ cells and Caspase3^+^CD5^+^CD69^+^CD4^+^CD8^+^ cells, as previously proposed (8, 31). No significant differences were observed in the proportions of the two cell populations: Caspase3^+^CD5^+^CD69^+^CD4^+^ (WT: 0.035 ± 0.011; EphB2^−/−^: 0.026 ± 0.008; EphB3^−/−^: 0.035 ± 0.013) and Caspase3^+^CD5^+^CD69^+^CD4^+^CD8^+^ (WT: 0.031 ± 0.009; EphB2^−/−^: 0.039 ± 0.008; EphB3^−/−^: 0.020 ± 0.004). Mutant mice living in non-sterile conditions did not show apparently immunological deficits (30) or any signs of autoimmunity since no substantial lymphoid infiltrates occur in their livers or salivary glands (unpublished data).

## T-Cell Development in an Altered Thymic Epithelium

Taken together, these results confirm that, except for increased proportions of mutant DN thymocytes and lymph node Treg cells, the percentages of immunocompetent cells do not vary significantly in EphB-deficient animals. Nevertheless, more functional, *in vivo* approaches are necessary to definitively determine the immunological conditions of EphB-deficient animals. By contrast, the TEC network is deeply altered in these mutants making it difficult to explain how these changes do not affect thymocyte development since T–TEC interactions are considered critical for functional maturation of T cells (32, 33).

Results on the effects of Eph/ephrins on thymocyte maturation are few and contradictory presumably reflecting different background of mutant mice, protocols used to evaluate effects of Eph/ephrins and/or specificity of molecules studied, as previously discussed (3). No anomalies have been described in mice with conditionally deleted EphB4 gene in TECs (34), deficiency in EphB6 (24), or in four Ephs, EphB1, B2, B3, and B6 (23). However, other authors have found poor *in vitro* responses of EphB6^−/−^ T cells after anti-CD3 plus anti-CD28 stimulation together as well as *in vivo* decreased hypersensitivity and autoimmune responses (35). On the other hand, the deletion of either ephrin-B1 or ephrin-B2 in thymocytes does not course with thymus phenotypes (36, 37) but the lack of two molecules results in alterations in thymocytes and thymic structure (38), and low sensitivity to different autoimmune models, including experimental autoimmune encephalomyelitis (39) and collagen-induced arthritis (40). We also observed decreased values of positive selected TCRαβ^hi^CD69^+^ thymocytes in ephrin-B1 and/or ephrin-B2 deleted in TECs, particularly when using outbred mouse strains. In this case, changes in these lymphoid subsets course with profound alterations in the histological thymus organization (41). In addition, the thymus of EphA4-deficient mice show reduced proportions of both DP TCRαβ^hi^ cells and CD69^+^ cells in correlation with the collapse of thymic cortex (42).

A first glance at these results could suggest a possible association with the pattern of Eph/ephrin expression. At the earliest stages of thymic development, when TEC predominates in T-cell numbers, the proportions of EphB2, EphB3, and ephrin-B2-expressing cells rise to the highest values, sharply declining later, except for ephrin-B2^+^ cells that remain high until E14.5. Furthermore, in these early stages, the proportions of Eph/ephrin B expressing TECs are significantly higher than those of eph/ephrin B^+^ thymocytes (12). It is tentative to speculate that a greater EphB-dependent effect could occur in cell types containing higher numbers of cells expressing these molecules.

Another important point is the condition of thymocyte-TEC contacts in EphB-deficient thymi. Many features of these thymi do not favor the establishment of such interactions. Thus, increased proportions of apoptotic TECs and the appearance of huge epithelial-free areas make difficult thymocyte-TEC contacts in EphB-deficient mice (22). The point is to determine whether these changes are sufficient to provoke severe deficits in the molecular communications between thymocytes and TECs that result in holes in the T-cell repertoire or, by contrast, whether the remaining unchanged epithelial areas expressing key molecules for thymic functional selection, such as β5t, Aire, MHCII, and CD80 are capable of supporting an efficient T-cell maturation. Some recent results relating to the number and cell composition of thymic nurse complexes (TNCs) in EphB-deficient thymi constitute an illustrative example of our hypothesis. Previously, we demonstrated impaired establishment of DP T–TEC conjugates derived from mutant thymi (13).

**Figure 2:**
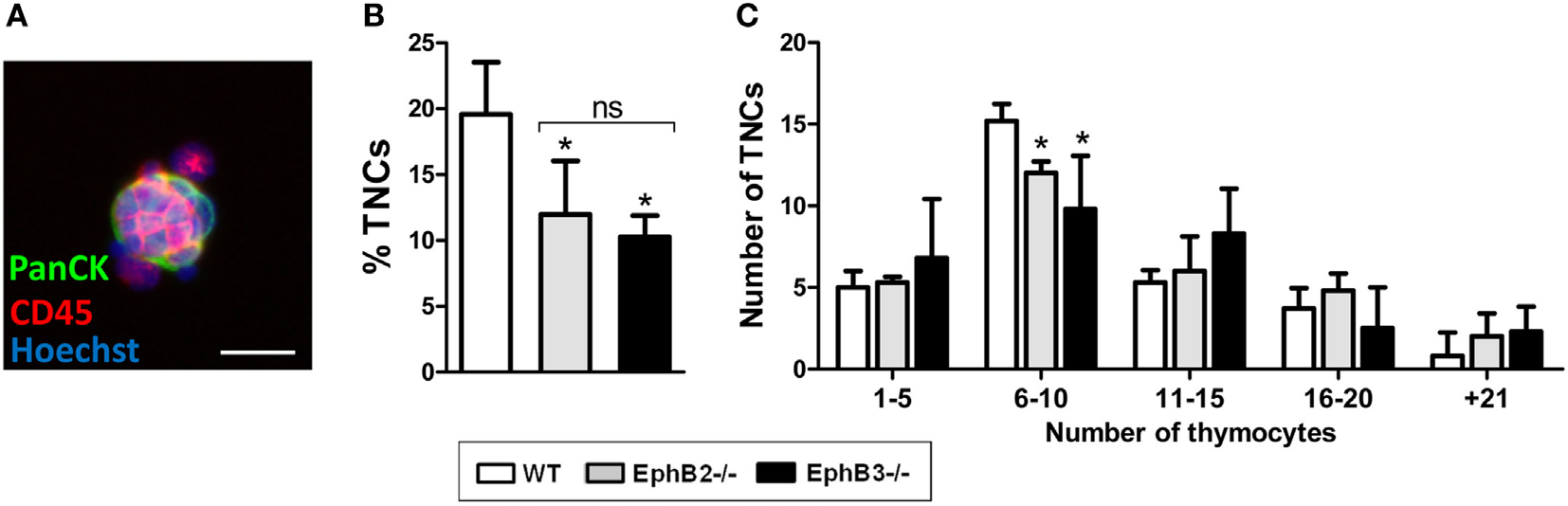
Analysis of thymic nurse complexes (TNCs) in adult WT and EphB-deficient thymi. **(A)** Representative thymic nurse complex formed by thymic epithelial cells, stained with an anti-PanCytokeratin antibody (PanCK, Green) and thymocytes identified by using an anti-CD45 antibody (Red). Nuclei are stained with Hoechst 33342 (Blue). Scale: 20 µm. **(B)** A significantly lower percentage of isolated TNC in EphB2^−/−^ and EphB3^−/−^ thymi than in WT ones. **(C)** According to the number of thymocytes included in the TNCs, six different groups could be established. The figure shows the TNC numbers of WT and EphB-deficient thymi after the analysis of 30 TNCs. In both WT and mutant thymi, the distribution is similar but the frequency of those containing 6–10 thymocytes, which represent the half of total TNC analyzed, is lower in mutant than in WT ones. The significance of the Student’s *t*-test probability is indicated as *p ≤ 0.05. ns: non-significant.

Thymic nurse complexes were first considered a kind of *ex vivo* specialized thymic microenvironment for T-cell maturation in which a single cTEC constituted lymphostromal complexes with 7–50 thymocytes (43, 44). We analyzed comparatively TNCs (Figure 2A) isolated from either WT or EphB-deficient thymi, confirming the expression in them of cTEC (i.e., Ly51, CD205, CD40) markers and MHCII, but not of MTS20 or MTS10 typical molecules of immature cells and mTECs, respectively. Both epithelial cells and thymocytes of nurse complexes also express EphB2, EphB3, and their ligands, ephrin-B1 and ephrin-B2, but the number of complexes yielded from mutant thymi was significantly lower than those from WT ones (Figure 2B). Most isolated TNCs contained 6–10 thymocytes and those composed of more than 21 thymocytes were the least represented. Mutant TNCs showed a similar range but exhibited significant reduced numbers of the most frequent ones containing 6–10 thymocytes (Figure 2C), suggesting that the lack of Eph/ephrin B affected the T–TEC interactions necessary to form the TNCs.

Although it is currently recognized that nurse complexes are special cortical areas involved in positive selection where long-lived DP thymocytes undergo secondary TCRα chain rearrangements (45), we failed to find changes in the proportions of positively selected TCRαβ^hi^CD69^+^ thymocytes indirectly suggesting that alterations found in TNCs from EphB-deficient thymi are not sufficient to impair positive selection.

These results demonstrate therefore that mutant TECs express all the molecules necessary to interact with developing thymocytes and to promote their proper differentiation, although their appearance and maturation is delayed with respect to the WT epithelium (12, Montero-Herradón et al 2017, submitted manuscript)^1^. In this respect, it has been claimed that just a few unaltered areas of thymic stroma could be sufficient to support a quite normal T-cell development (21, 46). Conditional deletion of Stat3 in K5^+^ TEC that courses with changes in medulla histology and decreased proportions of mature MHCII^hi^Aire^+^ mTECs does not affect autoimmune reactivity (47). More recently, specific deletion in TECs of the LTβR gene produces important changes in mTECs, including the disruption of typical 3D medulla organization with small, scattered medulla foci, and reduced numbers of mTECs^lo^, mTECs^hi^, and Aire cells (8), quite similar to the phenotype described for the thymic medulla of EphB-deficient mice. However, the frequencies of CD4^+^ and CD8^+^ thymocytes are unchanged, and the peripheral lymphoid organs of these mice are intact, suggesting that they do not undergo autoimmunity or exhibit an altered T-cell repertoire.

In agreement with our hypothesis, these authors conclude that despite limited numbers of tissue-related antigen-producing cells, the capacity of these mutant thymi for self-antigen production dependent on mTECs would exceed the threshold required for tolerance induction (8), explaining the absence of immune deficits.

## Ethics Statement

The study was carried out in accordance with the recommendations of the “Ethic Committee for Animal Research” of Complutense University. The protocols were approved by the Regional Government of Madrid.

## Author Contributions

JM, JG, and AZ have contributed to the design of the manuscript. SM, BS, and DA have provided technical results. All authors have read and accepted the final manuscript.

## Conflict of Interest Statement

The authors declare that the research was conducted in the absence of any commercial or financial relationships that could be construed as a potential conflict of interest.

## Footnotes

^1^Montero-Herradon S, Garcia-Ceca J, Zapata AG. Delayed maturation of mTEC in EphB-deficient thymi is recovered by RANK signaling stimulation. Manuscript submitted (2017).
